# How can health technology assessment be improved to optimise access to medicines? Results from a Delphi study in Europe: Better access to medicines through HTA

**DOI:** 10.1007/s10198-023-01637-z

**Published:** 2023-11-02

**Authors:** Anna-Maria Fontrier, Bregtje Kamphuis, Panos Kanavos

**Affiliations:** LSE Health—Medical Technology Research Group and Department of Health Policy, London School of Economics and Political Science, Houghton Street, London, WC2A 2AE UK

**Keywords:** Health technology assessment, HTA, Europe, Medicines, Access, Delphi, I, I1, I10, I11, I18

## Abstract

**Introduction:**

Access to medicines is a shared goal across healthcare stakeholders. Since health technology assessment (HTA) informs funding decisions, it shapes access to medicines. Despite its wide implementation, significant access variations due to HTA are observed across Europe. This paper elicited the opinions of European stakeholders on how HTA can be improved to facilitate access.

**Methods:**

A scoping review identified HTA features that influence access to medicines within markets and areas for improvement, while three access dimensions were identified (availability, affordability, timeliness). Using the Delphi method, we elicited the opinions of European stakeholders to validate the literature findings.

**Results:**

Nineteen participants from 14 countries participated in the Delphi panel. Thirteen HTA features that could be improved to optimise access to medicines in Europe were identified. Of these, 11 recorded a positive impact on at least one of the three access dimensions. HTA features had mostly a positive impact on timeliness and a less clear impact on affordability. ‘Early scientific advice’ and ‘clarity in evidentiary requirements’ showed a positive impact on all access dimensions. 'Established ways to deal with uncertainty during HTA’ could improve medicines’ availability and timeliness, while more ‘reliance on real-world evidence’ could expedite time to market access.

**Conclusions:**

Our results reiterate that increased transparency during HTA and the decision-making processes is essential; the use of and reliance on new evidence generation such as real-world evidence can optimise the availability of medicines; and better collaborations between regulatory institutions within and between countries are paramount for better access to medicines.

**Supplementary Information:**

The online version contains supplementary material available at 10.1007/s10198-023-01637-z.

## Introduction

Access to medicines is a multifaceted concept in that it is informed or influenced by different access dimensions, such as the availability of medicines within markets and the affordability of the healthcare system, among others. The Word Health Organisation (WHO) states that access to medicines is achieved when access is affordable and the medicines are safe, of high quality and effective [1]. The European Parliament (EP) has suggested that Europe should “*guarantee the right of patients to universal, affordable, effective, safe and timely access to essential and innovative therapies*” [2]. Even though better access to medicines might be a shared goal amongst healthcare stakeholders, its achievement has proven complicated. In Europe, a plethora of evidence showcases variability in access to medicines across countries [3–11]. These variations can be attributed to a variety of factors: some are associated with broader-level features such as (i) the general country characteristics, including gross domestic product (GDP) per capita and the epidemiological profile; and (ii) the country’s healthcare system characteristics, including healthcare expenditure, organisation of the healthcare system and clinical practices. Others are associated with more specific features such as (iii) the pharmaceutical market characteristics, including regulatory frameworks and the policies medicines undergo to become available and publicly funded in a given market [3]. Regulatory frameworks and policies are of particular interest to policymakers because they are amenable to policy changes. However, they can still be further complicated by the need to find a balance across different perspectives and objectives of involved stakeholders. For instance, whilst healthcare payers are seeking ways to optimise costs and ensure the sustainability of the healthcare system, patients seek timely access to medicines without considering the likely burden on local budgets.

In recent years, health technology assessment (HTA) has become one of the most important stages for efficacious and cost-effective medicines to become available and accessible to patients [12]. HTA recommendations play a crucial role in informing pricing and reimbursement decisions, facilitating negotiations, and updating national clinical guidance on disease treatment protocols, which can further impact the diffusion and uptake of new technologies [13–18]. Nowadays, HTA is used across all European countries, at least to some extent [13]. However, discrepancies are seen in the way HTA systems are set-up, the processes that are employed, the way assessment is performed, and the extent to which HTA recommendations inform reimbursement decisions, all of which can have an impact on access to medicines [5, 6, 8–11, 13, 15, 17, 19–30].

Within the European context and to alleviate access inequalities occurring due to variations in the conduct of HTA, numerous efforts have been made at both EU and national levels to harmonise, simplify, and expedite HTA processes [31–33]. Furthermore, efforts to establish collaborations between regulatory agencies and HTA bodies, such as parallel review processes and early scientific advice, are taking place to ensure that some alignment exists between what regulators and HTA agencies want, ultimately impacting patients’ access to the right treatment in a timely manner [16, 17, 25, 34]. However, evidence is scarce on what features of HTA, from the way it is set-up within the healthcare system to its role in funding decisions, are more likely to positively impact access to medicines beyond the details of submissions by manufacturers, including the clinical and economic evidence and their respective quality [8, 9, 17, 35–38]. Additionally, it is not clear whether current efforts aiming to improve HTA systems and processes, such as the harmonisation of clinical assessments through the new EU HTA regulation [31], are welcomed by both Western and Eastern European countries given differences in how well developed HTA processes are. And whether these efforts are considered as successful means to optimise access to innovative medicines by relevant stakeholders. Finally, evidence is scarce on what dimensions of access (e.g., availability, time to patient access, affordability) are targeted and, potentially, improved by different HTA features and components. In a nutshell, there is a gap in the literature on how HTA can be improved in a holistic way (i.e.: from its set-up to its uptake in funding decisions) to facilitate access to medicines across Europe and in light of the implementation of the new EU HTA regulation [31].

In addressing the above gaps, the objectives of this study are twofold: First, to explore how can HTA be improved to optimise access to medicines. And second, to assess levels of agreement between stakeholders from different geographic jurisdictions and/or different stakeholder groups on what features of HTA are more likely to have the most positive impact on access. To engage and elicit the views of European stakeholders, a Delphi exercise was conducted to develop an expert-based judgment [39]. Contrary to simple surveys and interviews, the Delphi method structures and organises group communications while allowing for controlled feedback [40–42].

While there are studies in the literature which use the Delphi method to elicit opinions on subjects such as value assessment of medical devices [43, 44], population health [45] and digital health technologies [46], to our knowledge there is only one study similar to ours in remit. This study explores how HTA for medicines can be improved across Europe, but with a different focus on the value assessment of oncology and haematology products, and the recent EU HTA regulation [33]. In our study, we aimed to validate HTA features that existing studies found to have an impact on access to medicines, and explored how a better understanding of these features through expert views can help improve HTA at national, regional and supranational levels in a holistic way (i.e.: from its set-up to its uptake in funding decisions) in order to facilitate access to clinically- and cost- effective medicines.

## Methods

Both primary and secondary evidence were used. Secondary data collection was conducted through a scoping review of the literature to identify, first, a list of HTA features that have shown to have an impact on access or features that could be improved. And second, to identify relevant access dimensions. Primary evidence was collected through a web-based Delphi panel in European stakeholders from both Western and Eastern European countries to validate the findings of the literature.

### Scoping review: HTA features and access dimensions

A scoping review was selected over a systematic literature review, as the scope of our search and the inclusion criteria were broader than the ones usually used in a systematic literature review. Generally, scoping reviews can help identify and map available evidence that is still unclear and cannot yet be addressed through a more precise systematic review [47].

#### HTA features

**Fig. 1:**
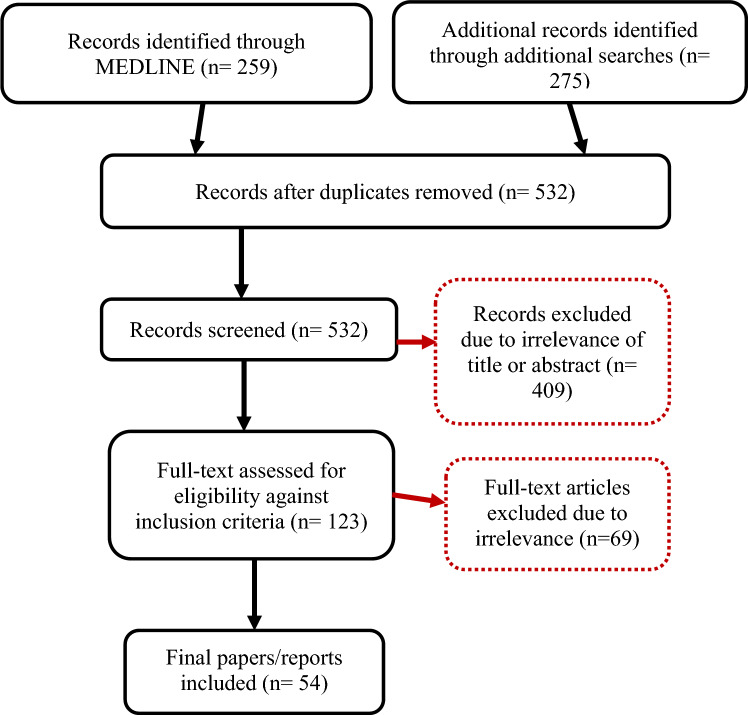
Flow diagram of the scoping review process

To identify recent peer-reviewed literature on HTA features and areas for improvement, we searched the MEDLINE via the PubMed database from January 2011 to December 2021 using the keywords (’health technology assessment’ OR ‘HTA’ OR ‘value assessment’) AND ‘Europe’. A detailed description of the scoping review strategy including the screening process and the exclusion and inclusion criteria used is outlined in detail in Appendix 1. The titles and abstracts of the resulting papers were screened by the first two authors in a double-blind fashion. Any disputes were resolved between first two authors. Papers considered relevant to our study objectives were downloaded and screened by the first author. An additional search was conducted by the first author on the websites of the European Commission and EUnetHTA to identify relevant grey literature using ‘Health technology assessment’ OR ‘HTA’ as key terms. Reports published from 2017 and onwards were included to capture recent developments and the current landscape of HTA in Europe. Figure 1 outlines the different steps and respective search results of the scoping review.

Relevant evidence was recorded and grouped into four main categories/endpoints, following an iterative process. The identified HTA features and components related to: (i) HTA system set-up; (ii) HTA procedures; (iii) HTA evaluation processes; and (iv) HTA and funding. An additional endpoint was created to record evidence on the access dimensions used in the relevant studies. The results of the scoping review on HTA features are summarised in Appendix 2. Table 1 presents the list of HTA features considered relevant in having an impact on access to medicines in the European region (Table 1).

**Table 1:** Features related to HTA as shown in the web Delphi panel

No	HTA features
HTA system	
1	Presence of an independent HTA body
HTA procedures	
2	Scientific advice (feedback and advice on upcoming applications) provided to manufacturers ahead of commencement of formal HTA process by HTA bodies
3	Introduction of parallel review process to streamline marketing authorisation and HTA
4	Stakeholder involvement during the HTA process
5	No reliance on “HTA referencing” (requirement for positive HTA recommendations from other countries to commence or conclude the HTA process or reliance on HTA recommendations from other countries to inform decision-making)
6	Agreed-upon timelines for the completion of HTA process
HTA evaluation processes	
7	Clarity of evidentiary requirements for value assessment in HTA (e.g., clear instructions published by the HTA body on the evidence to be submitted by manufacturers; evidentiary requirements based on a validated or publicly available framework)
8	Reliance on real-world evidence in HTA in case of limited, incomplete, immature, or early phase clinical evidence
9	Harmonisation of rules for HTA methodologies, evidentiary requirements, and procedures across HTA bodies and systems at supranational level
10	Coordination of HTA rules, methods and processes across national and regional level, if both co-exist
11	Explicit recognition of additional dimensions of benefit beyond clinical and/or economic evidence considered during the evaluation of health technologies (example dimensions include unmet medical need, impact on carers and family, impact on society, etc.)
12	Established procedures on how uncertainties resulting from submitted evidence are managed and resolved within an agreed-upon timeframe (e.g., request of additional evidence, sensitivity analysis, dossier re-submission)
HTA and funding decisions	
13	Legally binding HTA recommendations to be implemented in the shortest possible timeframe during reimbursement negotiations

#### Access dimensions

To provide a comprehensive definition of access to medicines, the different dimensions of access used in the resulting papers of the scoping review (described above) were explored, when available. Additional searches were conducted on the websites of international organisations such as the WHO, the United Nations and the European Commission, using the key term “access to medicines” OR “patient access” OR “access”.

Three relevant dimensions of access were identified and included in this study. The dimensions and definitions of access are used for the sole purpose of this study are as follows:

- Availability of medicines: whether clinically- and cost–effective medicines are available and marketed in a given market;
- Time to patient access (timeliness): the timely access of patients to publicly reimbursed medicines, and;
- Affordability: whether the prices of clinically- and cost- effective medicines are in line with the purchasing ability of healthcare systems and of patients.

### The Delphi process

The Delphi method can be used to fulfil a variety of research objectives such as reaching participant consensus on a complex topic, prioritisation of policies, and generation of debate among participants who might not share a common vision [48, 49]. The Delphi method can also be used when current knowledge is incomplete, uncertain or lacking [50]. During a series of rounds (surveys), panel participants can first respond to a set of questions and, in subsequent rounds, are given the opportunity to re-consider and re-assess their initial opinions after seeing the aggregate responses of other participants [40, 45, 48, 51–55]. Hence, the Delphi method is an iterative process that avoids intentional and unintentional noise, such as irrelevant and non-productive communication among the participants [42, 48]. Panel responses are always anonymous allowing participants to express their opinion freely without introducing potential bias due to peer pressure or the presence of potentially dominant or more vocal experts [40, 45, 48, 51–56].

No set minimum or maximum participant number for Delphi panels exists, with Delphi panels being conducted from five up to thousands of participants [39, 50, 57–62]; an appropriate number is most likely dictated by the objectives and nature of the research, though methodological advice, and Delphi panels in practice, often range between 10 and 20 participants [54, 60–63].

Even though Delphi panels may usually include three or more rounds to reach consensus amongst participants, in this study we deemed that two rounds were sufficient to ensure desirable completion rates, in line with other studies in the literature [39, 57, 58, 64]. This is because we had already compiled a list of HTA features likely to have an impact on access, thus an initial round soliciting experts’ opinions was deemed unnecessary.

### Stakeholder sample

A list of stakeholders was compiled from the authors’ network, considering their knowledge and areas of expertise, country of origin/work, and affiliation. Overall, our sample followed a purposive and snowball sampling strategy targeting experts in HTA from all European Union Member States, Norway, Switzerland, and the United Kingdom. Invited experts (*n* = 128) were either from academic or health policy research institutions, the pharmaceutical industry, decision-making/payer bodies, or patient organisations to capture the views of relevant stakeholders. To ensure a representative sample of European stakeholders, we invited a minimum of four experts, one of each stakeholder group, across all study countries. A limitation of this study is that healthcare professionals were not included in the sample as the authors were unable to identify clinicians that were familiar with and/or involved in HTA through either their network or the sampling strategies used.

### Study design and administration

The survey was piloted with five health economists from our institution to reflect on the structure and content prior to dissemination to external participants.

All stakeholders were invited via a personal email sent by the authors inviting them to participate in a two-round Delphi panel. Experts who indicated they were unable to participate were asked to identify a team member or colleague with similar expertise as a replacement. Where an alternate expert was identified, the original invitee was asked to provide the name, email and job title of their suggested colleague to ensure that their expertise was relevant to the research objectives of this study.

The study utilised a web platform, Welphi®, for the Delphi process. The platform ensures all experts received an automated email with a unique URL link. Participation was anonymised by Welphi® and each participant had a unique identifier containing an alphanumeric string (e.g.: 079AB). These identifiers allowed the authors to track whether the same individual participated in both Delphi rounds. Each round remained open for a month to accommodate schedules and availability. Automated reminders were sent every week to participants who had not started the survey and participants who had not yet completed their responses.

Participants were requested to complete an informed consent form to be able to continue with the Delphi process. All participants were asked to respond to demographic questions including the country they live and work in, their organisation affiliation, and their perspective selected from a list of pre-defined categories: research and policy, patient/patient organisation, industry, or decision-maker/payer. Participants were given clear definitions of all three access dimensions, and were able to rank their agreement using a five-point Likert scale (‘strongly agree’ (SA), agree’ (A), ‘neither agree nor disagree’, ‘disagree’ (D), ‘strongly disagree’ (SD)) on the positive impact of the HTA features on the three access dimensions. To ensure reliability of the panel’s outcomes, participants were given the option to select ‘do not know’ for instances where they did not feel confident about their response and a ‘not applicable’ option was also given to allow participants to indicate HTA features they felt might not be relevant to an access dimension. A single, open-ended question was available to the participants in the first round only to provide the opportunity to add any factor or HTA feature that, in their opinion, might have a positive impact on access and was not identified through our scoping review. However, these responses were used only as contextual information and were not included as statements in the second and final round of the Delphi panel for two reasons: first, the objective of the study was to validate the results of the scoping review and; second, if these new statements would have been included in the second round, the participants’ ability to engage with the statements would have been limited as they would have not been able to see the aggregate responses of the participants and potentially revise or keep their initial responses in an additional round, which is a main feature of the Delphi method.

In round 2, participants were asked to rank again the value statements. In this round, participants were able to see the aggregate responses of all the participants from round 1 as percentages. Participants had the option to revise or keep their initial responses from round 1. The study received ethics approval by our institution.

### Data analysis

The analytical methods employed were chosen considering the ordinal scale nature of our data, our study objectives and the results of a thorough search of the literature on Delphi panel methodologies [48, 51, 58, 59, 65–67] and other studies using the Delphi method [45, 60, 68–77]. Quantitative methods were used, including both descriptive and inferential statistics, to explore (i) what features of HTA had the most positive impact against different access dimensions in the final round; (ii) the level of agreement between stakeholders about the impact and rank of different HTA features across access dimensions in both rounds, and (iii) how stable their responses were across rounds. The open-ended responses provided by the participants in the first round were used only as contextual information and were excluded from the data analysis.

Different measures and methods were used to explore the aforementioned points which are outlined in detail in Table 2. For points (i) and (iii), additional analyses were performed using more than one commonly used method to validate the robustness of our results, recognising that there is limited to no evidence on which exact method is the most suitable to use in specific circumstances, or how results can change when using different methodologies. All analysis was conducted for 39 value statements (13 HTA features across the three access dimensions).

**Table 2:** Summary of definitions and methods used in this study

Definition(s)		Method	Interpretation
Agreement	The group agreement on the positive impact of HTA features on access dimensions in round 2^1^	Percentage agreement	Approved by absolute majority: SA > 50% and SD + D < 33.3% Qualified majority: SA + A > 75% Rejected by absolute majority: SD + D > 50% [45, 48]
		Central tendency and level of dispersion using median and the interquartile range (IQR)	Positive impact: median of 1 (SA) or 2 (A) No positive impact: median of 4 (D) or 5 (SD) Agreement: IQR ≤ 1 (i.e.: more than 50% of all opinions fall within 1 point on the scale) Lack of agreement: IQR > 1 [48, 70, 72, 77]
	The likelihood at which participants independently rate a given statement with the same rank in each round accounting for agreement occurring simply by chance /*Whether participants agree with each other on the ranking they gave for each value statement in each round*	Inter-rater reliability (IRR) using Gwet’s kappa coefficient applying ordinal weights	Poor agreement: Gwet’s kappa < 0.00 Slight agreement: 0.00 > Gwet’s kappa ≤ 0.20 Fair agreement: 0.20 > Gwet’s kappa ≤ 0.40 Moderate agreement: 0.40 > Gwet’s kappa ≤ 0.60 Substantial agreement: 0.60 > Gwet’s kappa ≤ 0.80 Almost perfect agreement: 0.80 > Gwet’s kappa ≤ 1 [83]
Stability and consistency	The stability/consistency of group responses per value statement between rounds/ *The likelihood participants changed their opinion/rankings as a group from round 1 to round 2*	Non-parametric Wilcoxon matched-pairs signed-rank test	Stable response: p value > 0.05 non statistically significant change Unstable response: p value ≤ 0.05 statistically significant change [48, 73, 84]
		Spearman’s rho coefficient	High degree of concordance: positive correlation coef. (*ρ* ≥ 0.75) with *p* value ≤ 0.05 showing that is statistically significant Low degree of concordance: negative correlation coef. (ρ < 0.75) with *p* value > 0.05 showing that is non-statistically significant [48, 72, 84]
Consensus^2^	*Consensus was considered achieved when a value statement was approved by qualified majority or had a median of 1 and 2 and IQR* ≤ *1 in round 2 and showcased stability (non-statistically significant change) between rounds*		

*Source:* The Authors based on a search of the literature on Delphi panel methodologies [48, 51, 58, 59, 65–67] and other studies using the Delphi technique [45, 60, 68–77]

^1^Group agreement has been calculated for both rounds. However, in the results section, we present the value statements that reached agreement in the 2^nd^ round. Appendix 2 includes results across all rounds

^2^Since this measure is subjective, it was used only for the purposes of the discussion section

Strongly agree (SA) and agree (A) and strongly disagree (SD) and disagree (D) responses were grouped, respectively, for the percentage agreement analysis. Median and interquartile ranges, rather than mean and standard deviation, were used for measuring central tendency and level of dispersion to avoid skewed results due to outliers. Gwet’s kappa coefficient was selected to test inter-rater agreement on each round over other kappa coefficients as it allows for multiple participants and any level of measurement by applying relevant weights for the ordinal scale, and missing values due to the selection of the ‘do not know’ or ‘not applicable’ options [78–81]. The ‘do not know’ and ‘not applicable’ responses were excluded from the quantitative analysis to limit analysis of agreement to participants who were confident in their responses.

Finally, since consensus is a term poorly and ambiguously defined in the literature [48] while its measurement greatly varies across studies [48, 50, 82], in this study, we differentiate between agreement and consensus. For consensus, stricter criteria were applied compared to group agreement to avoid inconclusive results. However, given that consensus is based on subjective criteria, it was only used for discussion purposes. All analyses were conducted in Stata SE 16.1 and SPSS Version 27.

## Results

### Participation rate

**Fig. 2:**
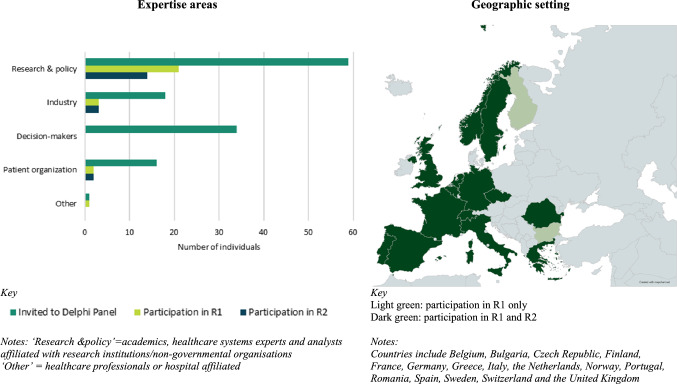
Expertise and geographic setting for participants in round 2

A total of 128 participants across Europe were approached for involvement in the Delphi panel. Of these, 27 participants from 16 European countries took part in round 1. From the 27 participants in round 1, 19 participants from 14 countries completed round 2. Figure 2 illustrates the characteristics of the stakeholders from round 2.

### Delphi panel results

We present the results of all statistical analyses across 39 value dimensions (13 HTA features across three access dimensions). Appendix 3 provides the results for both rounds.

### Agreement

#### Group agreement on value statements with the most positive impact on access dimensions in round 2

Table 3 summarises the group agreement on the positive impact of each HTA feature on each access dimension in round 2.

**Table 3:** Results of agreement in round 2 using percentage agreement, central tendency and median, and inter-rater agreement

HTA features	Availability				Time to patient access (timeliness)				Affordability			
	Agreement in round 2				Agreement in round 2				Agreement in round 2			
	% SA + A	median	IQR	Gwet's kappa (Level of agreement)	% SA + A	median	IQR	Gwet's kappa (Level of agreement)	% SA + A	median	IQR	Gwet's kappa (Level of agreement)
HTA system												
1	41%	3	1 *agreement*	0.59 *moderate*	47%	3	2 *no agreement*	0.06 *slight*	79%	2	0 *agreement*	0.42 *moderate*
HTA procedures												
2	89%	2	1 *agreement*	0.64 *substantial*	79%	2	1 *agreement*	0.52 *moderate*	79%	2	0 *agreement*	0.69 *substantial*
3	50%	2.5	2 *no agreement*	-0.07 *poor*	95%	2	1 *agreement*	0.53 *moderate*	32%	3	2 *no agreement*	0.09 *slight*
4	47%	3	1 *agreement*	0.08 *slight*	74%	2	1 *agreement*	0.64 *substantial*	63%	2	1 *agreement*	0.65 *substantial*
5	33%	3	1 *agreement*	0.59 *moderate*	35%	3	1 *agreement*	0.71 *substantial*	13%	3	0 *agreement*	0.71 *substantial*
6	63%	2	1.5 *no agreement*	0.38 *fair*	94%	2	0 *agreement*	0.72 *substantial*	12%	3	0 *agreement*	0.75 *substantial*
HTA evaluation processes												
7	76%	2	1 *agreement*	0.04 *slight*	95%	2	1 *agreement*	0.62 *substantial*	53%	2	1 *agreement*	0.50 *moderate*
8	88%	2	0 *agreement*	0.75 *substantial*	100%	2	0 *agreement*	0.47 *moderate*	37%	3	1 *agreement*	0.44 *moderate*
9	76%*	1	1 *agreement*	0.31 *fair*	89%	2	1 *agreement*	0.49 *moderate*	37%	3	2 *no agreement*	0.11 *slight*
10	75%	2	1.5 *no agreement*	0.37 *fair*	94%	2	1 *agreement*	0.57 *moderate*	53%	2	2 *no agreement*	0.02 *slight*
11	75%	2	1.5 *no agreement*	0.37 *fair*	72%	2	2 *no agreement*	0.24 *fair*	53%	2	1 *agreement*	0.46 *moderate*
12	94%	2	0 *agreement*	0.82 *almost perfect*	83%	2	0 *agreement*	0.78 *substantial*	61%	2	1 *agreement*	0.61 *substantial*
HTA and funding decisions												
13	65%	2	1 *agreement*	0.1 *slight*	76%	2	1 *agreement*	0.44 *moderate*	25%	3	1 *agreement*	0.67 *substantial*

SA + A: Strongly agree and agree; IQR: Interquartile range

Median: 1 = strongly agree; 2 = agree; 3 = neither agree nor disagree; 4 = disagree; 5 = strongly disagree

*This value statement was approved by absolute majority (SA > 50% and SD + D < 33.3%)

*Percentage agreement*: From a total of 39 value statements in round 2, 18 (46.2%) were approved by qualified majority (i.e.: SA + A > 75%), including one statement (‘*harmonisation of rules for HTA methodologies, evidentiary requirements, and procedures across HTA bodies and systems at supranational level’* on availability of clinically- and cost-effective medicines) which was approved by absolute majority (SA > 50% and SD + D < 33.3%). No value statement was rejected by absolute majority (SD + D > 50%), showing that there was no HTA feature in our list that many participants felt that it cannot have a positive impact on access.

Access dimensions: Most HTA features were found to have a positive impact on time to patient access (9 out of 13 HTA features). Seven HTA features were considered to have a positive impact on availability of medicines within markets while only two features were believed to have a positive impact on affordability for patients and healthcare systems.

HTA features: One HTA feature, ‘*scientific advice provided to manufacturers by HTA bodies ahead of the initiation of the HTA process*’, was considered to have a positive impact by qualified majority across all three access dimensions (89% on availability and 79% on both timeliness and affordability, respectively). ‘*Reliance on real-world (RWE) evidence in cases of limited clinical data*’ was the only HTA feature that all stakeholders (100%) believed to have a positive impact on time to patient access.

*Central tendency and level of dispersion*: 22 (out of 39) value statements reached agreement across participants, with a median of 1 or 2 and IQR ≤ 1 (56.4%) in round 2.

Access dimensions: Participants agreed (median:2 and IQR ≤ 1) that most HTA features had a positive impact on time to patient access (10 out of 13 HTA features with a median of 1 and 2 and IQR ≤ 1), while six HTA features resulted in agreement on their positive impact on availability. Six HTA features were found to have a positive impact on affordability.

HTA features: Participants strongly agreed (median:1; IQR:1) on the positive impact on availability of ‘*harmonisation of rules for HTA methodologies, evidentiary requirements, and procedures across HTA bodies and systems*’.

#### Factors not captured by the scoping review that might have an impact on access as suggested by participants in round 1

*Open-ended question*: Only three participants provided factors that could potentially have an impact on access not identified through the scoping review through a response to the open-ended question in round 1. These suggested factors included: (i) choosing a cost-effectiveness approach rather than comparative clinical benefit assessment; (ii) having pre-defined criteria for which stakeholders should be involved during HTA processes (for impact on availability, not necessarily time to access), and; (iii) having a linkage between horizon scanning, budgeting and HTA. These statements were not validated by the Delphi participants in the second round.

#### Overall group agreement per value statement in rounds 1 and 2

*Inter-rater reliability (IRR), Gwet’s kappa coefficient*: In round 1, low levels of agreement were observed across participants. Participants had fair or moderate agreement for 30.8% (12 out of 39) and 38.5% (15 out of 39) value statements, respectively. Substantial agreement was reached in only 15.4% (6 out of 39) of value statements, two of which had been approved by qualified majority and reached agreement through central tendency and low level of dispersion in round 1. There was no value statement with almost perfect agreement.

In round 2, agreement levels changed; 33.3% of value statements (13 of 39) resulted in substantial agreement and one value statement (on the positive impact of ‘*establishing procedures to deal with clinical and economic uncertainties*’ on availability) reached an almost perfect agreement. Six of the value statements showcasing substantial agreement amongst participants and the one value statement with almost perfect agreement were also approved by qualified majority and showcased a median of 1 or 2 with IQR ≤ 1 in round 2.

Table 3 summarises the overall group agreement per value statement in round 2.

### Stability and consistency of responses between rounds

*Non-parametric Wilcoxon matched-pairs signed-ranks test*: 94.9% of the value statements were stable between rounds (i.e.: not significantly changed). Only two value dimensions had a *p*-value less than 0.05 which indicated that they were statistically significant, thus unstable: these two were the positive impact of ‘*agreed timelines for the conduct of HTA processes’* on time to patient access, and the positive impact on the ‘*use of established procedures to handle uncertainty*’ on affordability.

*Spearman's rank-order correlation coefficient (Spearman's rho)*: Participants’ opinions had a statistically significant high degree of concordance in 69.2% (27 out of 39) of the value statements.

Table 4 presents the results of stability between rounds 1 and 2.

**Table 4:** Results of stability between rounds 1 and 2

	Availability		Time to patient access (timeliness)		Affordability	
HTA features	Stability between rounds					
	Spearman's rho (Level of concordance)	Wilcoxon matched-pair signed rank test (p-value)	Spearman's rho (Level of concordance)	Wilcoxon matched-pair signed rank test (p-value)	Spearman's rho (Level of concordance)	Wilcoxon matched-pair signed rank test (p-value)
HTA system						
1	0.97 *high degree*	*1.00* *stable*	0.91 *high degree*	0.50 *stable*	0.94 *high degree*	*stable*
HTA procedures						
2	0.94 *high degree*	0.50 *stable*	0.97 *high degree*	1.00 *stable*	0.85 *high degree*	1.00 *stable*
3	0.96 *high degree*	1.00 *stable*	0.96 *high degree*	0.50 *stable*	0.90 *high degree*	1.00 *stable*
4	1.00 *high degree*	1.00 *stable*	0.82 *high degree*	1.00 *stable*	0.92 *high degree*	1.00 *stable*
5	0.71 *low degree*	0.06 *stable*	0.52 *low degree*	0.17 *stable*	-0.03 *low degree*	1.00 *stable*
6	0.71 *low degree*	0.11 *stable*	0.49 *low degree*	0.00 *unstable*	0.20 *low degree*	1.00 *stable*
HTA evaluation processes						
7	0.93 *high degree*	0.50 *stable*	0.87 *high degree*	1.00 *stable*	0.79 *high degree*	0.25 *stable*
8	0.76 *high degree*	1.00 *stable*	0.77 *high degree*	0.50 *stable*	0.92 *high degree*	0.50 *stable*
9	0.99 *high degree*	1.00 *stable*	0.92 *high degree*	0.50 *stable*	0.98 *high degree*	1.00 *stable*
10	0.98 *high degree*	1.00 *stable*	0.96 *high degree*	1.00 *stable*	0.98 *high degree*	1.00 *stable*
11	0.83 *high degree*	0.50 *stable*	0.77 *high degree*	0.50 *stable*	0.99 *high degree*	1.00 *stable*
12	0.38 *low degree*	0.40 *stable*	*0.47* *low degree*	0.13 *stable*	0.55 *low degree*	0.02 *unstable*
HTA and funding decisions						
13	0.66 *low degree*	1.00 *stable*	0.16 *low degree*	0.27 *stable*	0.51 *low degree*	*0.06* *stable*

## Discussion

Using the Delphi method, we explored how HTA systems, procedures and processes can be improved to optimise access to medicines by canvassing opinions and perspectives of European HTA experts. Our results have several implications for both the HTA features and the access dimensions. However, they should be interpreted with caution due to the inherit limitations of the Delphi method, such as low participation and high dropout rates. In our study, a small number of experts participated in both rounds, and responses were predominately received from research and policy makers, with no opinions from healthcare professionals and decision-makers captured.

With regards to HTA features, 11 out of the 13 showed a positive impact on at least one of the three access dimensions suggesting that participants’ views are broadly aligned with current efforts and discussions on how HTA can be designed or adjusted at regional, national and supranational levels to optimise access to medicines. ‘Early scientific advice’ and ‘clarity in evidentiary requirements’ reached consensus on their positive impact on all access dimensions. Interestingly, even though many well-established HTA bodies in Europe currently provide early scientific advice to manufacturers and have published guidelines for evidence requirements, a call to action for some HTA bodies to (i) emphasise more the provision of early support to manufacturers before HTA initiation, (ii) provide more clarity on the evidence required for evaluation, and (iii) be more transparent and systematic on the way they deal with uncertainty if it arises, was identified.

‘*Established ways to deal with potential uncertainty occurring during HTA assessments’* reached consensus on its positive impact on both availability and time to patient access. This HTA feature was also identified by a recent study [33] which highlighted that the management of uncertainty is one of the challenges that need to be addressed to provide an ‘additional benefit’ to a European HTA process.

‘*Reliance on RWE in HTA’* reached 100% agreement among participants in the second round on its positive impact on timeliness, emphasising the importance of the use of new types of evidence beyond strict clinical studies which do not test for the clinical benefit of a medicine in a real-world setting. This has been extensively discussed across Europe, especially at regulatory and HTA levels for instances where clinical evidence might still be incomplete or of low quality. However, the use of RWE varies across countries with some HTA bodies accepting RWE while others do not [85], and with other access implications arising due to a lack of systematic ways to collect, interpret and use these data during assessments [86].

Looking at the results of all the analytical methods used and recognising that different methods can lead to different conclusions, we can only conclude confidently (SA + A > 75%, median:1 or 2 and IQR ≤ 1, substantial or almost perfect agreement and high degree of concordance and stable responses between rounds) that participants agreed on the positive impact of ‘*reliance on RWE’* on availability of medicines and of ‘*provision of scientific advice’* on both availability and affordability and; of ‘*clarity of evidentiary requirements for value assessment’* on timeliness.

Table 5 summarises the HTA features with the most positive impact on the respective access dimensions.

**Table 5:**
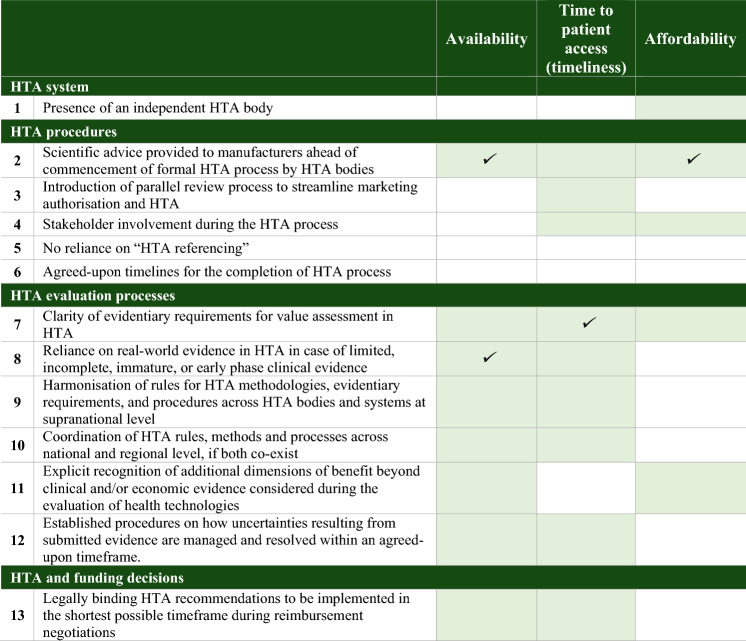
HTA features with the most positive impact on access dimensions

Notes: Green coloured cells show the HTA features that reached consensus on having the most positive impact on the respective access dimension (approved by qualified majority and/or having a median of 1 or 2 with low level of dispersion (IQR ≤ 1) and stable responses between round 1 and 2)

✔show the HTA features that we can confidently conclude that they have a positive impact on the respective access dimensions according to all the analytical methods used (SA + A > 75%, median:1 or 2 and IQR ≤ 1, substantial or almost perfect agreement of participants and high degree of concordance and stable responses between round 1 to 2)

Across the grouping of HTA features presented in Table 1, all features targeting evaluation processes reached consensus on their positive impact on at least two access dimensions: participants agreed access to medicines could be ameliorated by having clear guidance on what evidence is required, on ways to deal with uncertainty, and on the incorporation of additional dimensions of value beyond clinical and cost-effectiveness, together with general coordination and harmonisation of evaluation processes at regional, national and supernational levels. The new HTA regulation of the European Commission on joint clinical assessments across European Member States, to be officially implemented in 2025, aims to address access issues arising due to discrepancies in the evaluation processes of national/regional HTA bodies. The importance of this is also reiterated in our findings, as ‘*harmonisation of rules for HTA methodologies, evidentiary requirements, and evaluation procedures across HTA bodies and systems at supranational level*’ was approved by absolute majority in both rounds for its positive impact on the availability of medicines and reached consensus on its positive impact on availability and time to patient access. Therefore, standardising HTA evaluation processes and creating coherent and consistent scientific evidence collection, generation and interpretation across Europe could achieve better and more controlled access to medicines within countries. On the other hand, HTA features related to procedures and set-up reached consensus mainly on their positive impact on time to patient access and affordability, rather than the availability of medicines. As both of these are more relevant to the specificities of each setting, they should still remain a country competence taking into account country-specific characteristics, objectives and values and further reflect the way the healthcare system is organised [13, 16, 34].

With regards to the access dimensions, Delphi participants believed that the included HTA features mostly had a positive impact on timely access to publicly funded medicines which is in line with broader HTA objectives as a tool informing reimbursement decisions within nations or healthcare systems to streamline national/regional accessibility to medicines after receiving marketing authorisation. However, a number of concerns have been raised previously that HTA processes can hinder timeliness due to assessment delays and the presence of an additional regulatory step to medicines’ availability within markets [8, 87, 88]. More HTA features were expected to have a positive impact on affordability of the healthcare system, as HTA processes are implemented in an effort to allocate resources efficiently considering evidence-based information, the sustainability of the healthcare system, and the finite budgets available. On the contrary, HTA features with the lowest percentage agreement on their positive impact were identified on the affordability dimension. And interestingly, ‘*legally binding HTA recommendations for reimbursement decisions and/or negotiations’* did not reach agreement or consensus amongst participants in round 2, in having a favourable effect on affordability, even though required translation of HTA recommendations into funding would mean that the most cost-effective medicine would be covered using publicly available budgets.

Our findings on affordability, however, are not conclusive because of the lack of representation of decision-makers/payers in our sample. Yet, these findings can still observe what other HTA experts believe: For instance, the ‘*presence of an independent body’* reached consensus on its positive impact on affordability (and not on any other access dimension), highlighting that transparency and conflict of interest concerns may remain when HTA processes are integrated within national/regional healthcare payers/-decision-makers opposed to taking place independently at arm’s length [13]. Therefore, more transparency might be needed to better understand how HTA recommendations are used during negotiations and price setting within jurisdictions. However, this may only apply in some cases, as HTA systems for medicines integrated to governmental institutions are rarely seen in Europe [13].

Overall, even though HTA is an essential instrument to streamline and monitor access to medicines across settings, it is important to highlight that any action to achieve better and faster patient access should be complemented by other appropriate and effective regulatory policies and procedures, which are equally important. Targeted efforts and interventions in HTA alone will not necessarily translate to better patient access without adjustments in other areas: for example, if reimbursement policies are not adjusted to align to, or at least take into consideration, HTA recommendations which promote the most cost-effective therapeutic option. Not only should each stage of the access pathway aim to maximise the effects on improving access, it may also benefit from synergies between these stages. For instance, the ‘*introduction of parallel review processes’* reached consensus on its positive impact on time to patient access, highlighting that collaboration between marketing authorisation and HTA bodies could improve timeliness.

### Study limitations

Our study is not without limitations. First, our results should be interpreted with caution due to the small sample size caused by low participation and high dropout rates, limited or lack of representation of some stakeholder groups (i.e., healthcare professionals and decision-makers), and limited geographical representation. Additionally, participant representation which was skewed towards policy and research experts could have introduced bias in our results. Nevertheless, the findings of this study can still be considered informative in (i) identifying how different HTA features target different access dimensions, (ii) understanding (dis-) agreement on whether current efforts to improve HTA are successful according to experts from different geographic settings, and (iii) identifying areas of HTA that might need improvement, as long as, this limitation is acknowledged in the interpretation of these three conclusions. Second, while a scoping review was conducted to create a list of HTA features that might have an impact on access, this list may not be exhaustive. To address this, participants had the opportunity to respond to an open-ended question in round 1 to share additional HTA features that might have not been included in our list. Third, our Delphi panel included two rounds rather than three rounds. However, we deemed that two rounds were sufficient as we had already conducted a scoping review and compiled a list of HTA features that were likely to have an impact on access. Finally, there are numerous definitions for agreement, stability and consensus in the literature, which are often unclear, and each of these can rely on several different methodologies for results analysis. To address this, our study defined the relevant terms in detail and conducted analysis using more than one method, when applicable and appropriate.

## Conclusion

Using the Delphi method, this study found that improved HTA processes and procedures were shown to have a predominantly positive impact on timeliness, and a less clear impact on affordability despite HTA’s remit to ensure efficient allocation of finite resources. The most positive impact on all three access dimensions was seen on HTA features related to more clear, consistent and harmonised evaluation processes within and across countries, which is in line with current European efforts targeting the harmonisation of clinical assessment processes. Even though our results might not be conclusive, they reiterate the following overarching themes: increased transparency during HTA and decision-making processes is essential, use of and reliance on RWE can optimise availability of medicines, while better collaborations between regulatory institutions within and between countries are paramount for better access to medicines.

### Supplementary Information

Below is the link to the electronic supplementary material.

Supplementary file1 (DOCX 79 KB)
