# Supplementary material for: How can health technology assessment be improved to optimise access to medicines? Results from a Delphi study in Europe: Better access to medicines through HTA
Source: Eur J Health Econ. 2023 Nov 2;25(6):935–50. doi: 10.1007/s10198-023-01637-z (PMC11283424; doi:10.1007/s10198-023-01637-z)
Supplement: Supplementary file 1 — Supplementary file1 (DOCX 79 KB) [file 10198_2023_1637_MOESM1_ESM.docx]

**Appendix 1: Scoping review strategy to identify HTA features and areas for improvement to optimise access to medicines**

Our objective was to identify features and components of HTA that impact access to medicines favourably or unfavourably, or areas for improvement.

An advanced search was conducted in the MEDLINE via PubMed database to identify peer-reviewed papers from January 2011 to December 2021. The following keywords were used: (’health technology assessment’ OR ‘HTA’ OR ‘value assessment’) AND ‘Europe’. The relevant terms were searched through the titles and abstracts of the papers and the search was limited to the English language. Initially, ‘access’ was used as an additional key term, however, we removed it from the search terms as it was limiting our results substantially.

Search results were downloaded using EndNote and duplicates were removed when identified. An initial screening through the titles and abstracts was conducted by the first author to identify relevant papers that focuses on HTA of in-patent medicines and study access variations due to HTA. An additional screening in all the resulted titles and abstracts was conducted by the second author. Papers were excluded from the initial screening, when they were focusing on medical devices or other technologies (i.e.: vaccines, diagnostics, biomarkers or surgical procedures) or off-patent and generic medicines or hospital-based HTA; when they were clinical and cost-effectiveness assessments conducted by the authors instead of relevant national or regional authorities which officially conduct HTA; when alternative value assessment processes than HTA were explored, such as multiple-criteria decision analysis and; when other stages of a medicine’s access pathway such as regulatory and MA processes, the development phase of medicines or innovative pricing mechanisms and managed entry agreements or clinical guidelines, were studied.

The full texts of the remaining papers were screened by the first author, to identify features of HTA that facilitate or impede access or are responsible for observed variations in HTA recommendations for the same medicines across settings. Any information on current efforts to improve HTA processes at national or supranational level in Europe was also extracted. Any disputes on the inclusion of full-text, were resolved by the first two authors.

An additional search was conducted by the first author on the website of the European Commission and EUnetHTA to identify relevant grey literature on HTA in Europe. ’Health technology assessment’ OR ‘HTA’ were used as key terms for the search and the same inclusion and exclusion criteria as the ones used in the scoping review were used for the screening of the relevant hits. Reports published from 2017 and onwards were only included to capture recent developments and the current landscape of HTA in Europe.

Relevant information from both searches was recorded in an excel spreadsheet. Information was organised following an iterative process. Therefore, the extracted evidence was classified into the following four main categories/endpoints:

(1) ***HTA system,*** which included features related to how HTA is set up within the healthcare system;

(2) ***HTA procedures,*** which related to administrative stipulations of HTA;

(3) ***HTA evaluation process,*** which related to the assessment of the submitted evidence, and;

(4) ***HTA and funding decisions***, which reflected how and to what extent HTA is being used during funding decision-making.

Initially, information was recorded as a full text extracted from the initial source but was subsequently re-worded as statements that could improve access to medicines after three reiterations by the authors.

An additional study endpoint was used identify different access metrics. First, relevant evidence was extracted from the included studies of the scoping review. This information was further complemented with evidence from additional searches on the websites of international organisations. Through this endpoint, we were able to define access to medicines using different key metrics.

**Appendix 2: Scoping review results on HTA features and areas for improvement to optimise access to medicines**

**System set-up and organization.** The way healthcare and HTA systems are organised and set up can contribute to access delays (1–8). This can manifest itself within the HTA system, such as in Greece, where HTA is integrated within the government and the national payer and unclear or non-transparent connections and interactions between the multiple institutions involved result in unnecessary delays in funding negotiations (3,5), or in Italy, where the multi-level structure of HTA results in increased inequality of access to new medical technologies (6). Characteristics of the wider healthcare system may also create variation in patient access, such as the decentralised healthcare systems of Italy or Spain which can result in variation across regions because of divergent HTA recommendations or funding decisions due to differences in the methodologies used to assess technologies and the selection of technologies undergoing assessment (1,2,7–9).

**HTA procedures.** Extensive evidence variations in the HTA procedures employed by different HTA bodies, (such as whether HTA commences before or after marketing authorisation, the actual timelines of HTA evaluations, and whether and/or to what extent external stakeholders are involved in the HTA process) might result in access delays or create unnecessary access hurdles (3,8,10–18). An example of how HTA processes can impact access negatively is seen in a few European countries: Bulgaria and Romania both rely on HTA decisions of other well-established HTA agencies, and as such, HTA processes in these countries might be delayed until HTA recommendations are published in the countries they use as a reference (19). Or, how timelines for HTA recommendations to be published after marketing authorisation vary significantly across countries in practice: Spain had the longest timelines (mean time of 713 days), followed by Italy and Poland (504 and 462 days respectively), while France and Germany were the fastest, publishing HTA recommendations in 227 days and 199 days on average after marketing authorisation (2). Similar results were seen in another study where France had the fastest timelines (155 days) and Italy had the slowest ones (375 days) (12). When looking at the median timelines between MA and HTA submission, timelines also differed with seven days seen on average in England, 23 days in Italy, 29 days in France, 42 days in Germany and 49 days in Spain (12). These variations in time are due to different stipulations of HTA procedures. For instance, in Germany, the HTA assessment must be initiated within three months from MA approval according to German law (12). However, in other settings, HTA processes can only be initiated by manufacturers upon dossier submission (20). Stop-the-clock mechanisms, allowed by the English HTA for some medicines, can further lead to longer evaluation processes (12,21).

Better cooperation between regulatory and HTA bodies seems to be crucial for optimal and timely market access, with some collaborative processes being implemented in some settings to support this (22–26). The presence of early scientific advice from the HTA body to manufacturers before dossier submissions or during the medicine development process is a way to expedite HTA assessments (12,22–28). The provision of early scientific advice has aided manufacturers to generate evidence that meets the standards of both regulatory agencies and HTA bodies, however, according to key stakeholders this initiative had not yet succeeded to align regulatory and HTA requirements (28). Despite efforts to expedite assessment processes at regulatory level and harmonise assessment processes at both levels, still, a proportion of approved medicines do not result in positive HTA recommendations, and only in a few cases, HTA bodies seem to accept a lower quality of evidence which has already been approved by regulatory agencies (2,7,12,15,29–32).

Other elements which may have a positive impact is the stakeholder involvement in the HTA process (i.e.: for selecting which technologies should be assessed by the HTA body, during technology assessment or in the decision-making process for issuing HTA recommendation) (3,4,13,20,33–35). However, some researchers mentioned that divergent HTA recommendations for the same medicine across HTA bodies could be attributed to differences in the interpretation of the assessed evidence due to varying levels and types of stakeholders’ involvement (17,20,36–38).

**HTA evaluation processes.** Differences in HTA recommendations across HTA bodies or observed access delays can also be attributed to variations in the assessment practices followed by HTA bodies such as differences in evidentiary requirements, the potential inclusion of other value dimensions beyond clinical and cost-effectiveness, divergent ways to deal with uncertainty, and acceptance of real-world evidence (2,7,8,10–14,17,23,26,36,37,39–42,42–46). For example, HTA evaluation in Romania is mainly focused on costs rather than other additional value criteria creating challenges for patient access to innovative medicines (47), while in England a number of value and end-of-life criteria are considered explicitly in the assessment of some medicines, together with recognition of elements such as medicine innovation in deliberations on whether to accept higher willingness to pay thresholds (34,48). However, a study looking at 29 jurisdictions reported that more similarities than differences exist between major methodological aspects used in the HTA processes of the study countries, showing room for better cross-country co-operations (45). Another study reported, though, that manufacturers had to generate local contextualised evidence, including evidence on the local comparator, to meet specific evidentiary requirements of European HTA bodies. Almost 90% of submissions in England incorporated local information such as local standard of care and clinical practice, followed by 82% of the submission in Germany, 80% in Italy, 79% in Spain and 72% in France (12). Similarly, another study discussed that country-specific practice-related factors can explain differences in HTA recommendations across settings (16).

Heterogeneity in HTA recommendations across countries might also be attributed to differences in the acceptance of evidence from observational studies and indirect comparisons (37,41,42,45,49–51). However, evidence on whether acceptance of real-world data might improve access at HTA level is not widely positive: one study discussed that generation of real-world evidence might be one of the contributing factors for longer delays of access, as setting up registries can be time-consuming and bureaucratic (14), and another study highlighted that in Bulgaria, limited epidemiological data may pose an additional challenge to manufacturers for the preparation and submission of pharmacoeconomic and HTA dossiers beyond the lack of expertise for gathering data from real-world evidence (47).

**HTA and funding.** The relationship between HTA recommendations and funding decisions might play an important role for patient access. The most important challenge for patient access is a lack of an explicit framework on how to use HTA evidence in the decision-making process, while the availability of such a framework is among the most important facilitators (52). Evidence from middle-income European countries showed a lack of a legal framework for the implementation of HTA recommendations in funding decision-making (53), while another study highlighted that this phenomenon is also observed in high-income countries where HTA systems are well-developed and established (38).

Across Europe, there are countries where HTA recommendations are not legally binding and, thus, not always followed during decision-making processes (3,8,20,38). A study looking at the agreement between HTA recommendations and funding decisions for oncology medicines in Central and Eastern Europe showed that there was a low level of agreement between HTA and funding in Poland where HTA recommendations are non-binding, contrary to Latvia where recommendations are binding (19). Similarly, a study in Poland, where HTA recommendations are not legally binding, showed only a fair agreement between national HTA recommendations and ministerial funding decisions between 2013 and 2015 (54). A more recent study focusing on oncology medicines in Central and Eastern Europe showed that there was a low level of agreement between HTA and funding in Poland, contrary to Latvia where recommendations are binding (19). Therefore, in systems where HTA recommendations are binding, their implementation in funding decisions is more straightforward. For instance, the English NHS should reimburse and make available within a timeframe of three months a medicine that received a positive HTA recommendation by the English HTA body (7).

**References**

1. Martinalbo J, Bowen D, Camarero J, Chapelin M, Démolis P, Foggi P, et al. Early market access of cancer drugs in the EU. Annals of Oncology. 2016;27(1):96–105.

2. Akehurst RL, Abadie E, Renaudin N, Sarkozy F. Variation in health technology assessment and reimbursement processes in Europe. Value in Health. 2017;20(1):67–76.

3. Fontrier AM, Visintin E, Kanavos P. Similarities and Differences in Health Technology Assessment Systems and Implications for Coverage Decisions: Evidence from 32 Countries. PharmacoEconomics-Open. 2021;1–14.

4. Wilsdon T, Fiz E, Haderi A. A comparative analysis of the role and impact of health technology assessment: 2013. Washington DC: Charles River Associates. 2014;

5. Kanavos P, Tzouma V, Fontrier A, Souliotis K. Implementing health technology assessment (HTA) in Greece: Myths, reality and cautionary tales. Arch Hellen Med. 2019;37:444–51.

6. Ciani O, Tarricone R, Torbica A. Diffusion and use of health technology assessment in policy making: what lessons for decentralised healthcare systems? Health Policy. 2012;108(2–3):194–202.

7. Allen N, Walker SR, Liberti L, Salek S. Health technology assessment (HTA) case studies: factors influencing divergent HTA reimbursement recommendations in Australia, Canada, England, and Scotland. Value in Health. 2017;20(3):320–8.

8. Chamova J, Stellalliance A. Mapping of HTA national organisations, programmes and processes in EU and Norway. Publications Office of the European Union Luxembourg; 2017.

9. Mammarella F, Tafuri G. Innovative medicinal products: the new criteria of the Italian Medicines Agency. Recenti Progressi in Medicina. 2018;109(5):261–2.

10. Ades F, Zardavas D, Senterre C, De Azambuja E, Eniu A, Popescu R, et al. Hurdles and delays in access to anti-cancer drugs in Europe. ecancermedicalscience. 2014;8.

11. Bergmann L, Enzmann H, Broich K, Hebborn A, Marsoni S, Goh L, et al. Actual developments in European regulatory and health technology assessment of new cancer drugs: what does this mean for oncology in Europe? Annals of oncology. 2014;25(2):303–6.

12. Wang T, McAuslane N, Liberti L, Gardarsdottir H, Goettsch W, Leufkens H. Companies’ health technology assessment strategies and practices in Australia, Canada, England, France, Germany, Italy and Spain: an industry metrics study. Frontiers in Pharmacology. 2020;2017.

13. Angelis A, Lange A, Kanavos P. Using health technology assessment to assess the value of new medicines: results of a systematic review and expert consultation across eight European countries. The European Journal of Health Economics. 2018;19(1):123–52.

14. Şaylan M, Dokuyucu Ö. 14. Market Access Hurdles in Developed Countries. 2018;

15. Wang T, McAuslane N, Gardarsdottir H, Goettsch WG, Leufkens HG. Building HTA insights into the drug development plan: Current approaches to seeking early scientific advice from HTA agencies. Drug Discovery Today. 2021;

16. Vreman RA, Mantel-Teeuwisse AK, Hövels AM, Leufkens HG, Goettsch WG. Differences in health technology assessment recommendations among European jurisdictions: the role of practice variations. Value in Health. 2020;23(1):10–6.

17. Panteli D, Eckhardt H, Nolting A, Busse R, Kulig M. From market access to patient access: overview of evidence-based approaches for the reimbursement and pricing of pharmaceuticals in 36 European countries. Health research policy and systems. 2015;13(1):1–8.

18. Habl C, Laschkolnig A, Habimana K, Stürzlinger H, Röhrling I, Bobek J, et al. Study on impact analysis of Policy Options for strengthened EU cooperation on Health Technology Assessment (HTA). 2017;

19. Malinowski KP, Kawalec P, Trąbka W, Sowada C, Petrova G, Manova M, et al. Health technology assessment and reimbursement policy for oncology orphan drugs in Central and Eastern Europe. Orphanet journal of rare diseases. 2020;15(1):1–13.

20. Barnieh L, Manns B, Harris A, Blom M, Donaldson C, Klarenbach S, et al. A synthesis of drug reimbursement decision-making processes in organisation for economic co-operation and development countries. Value in Health. 2014;17(1):98–108.

21. Kristensen FB. Mapping of HTA methodologies in EU and Norway [Internet]. European Commission. 2017. Available from: https://health.ec.europa.eu/system/files/2018-01/2018_mapping_methodologies_en_0.pdf

22. Balaisyte L, Joos A, Hiligsmann M. Early dialogue in Europe: perspectives on value, challenges, and continuing evolution. International Journal of Technology Assessment in Health Care. 2018;34(5):514–8.

23. Bloem LT, Vreman RA, Peeters NW, Hoekman J, van Der Elst ME, Leufkens HG, et al. Associations between uncertainties identified by the European Medicines Agency and national decision making on reimbursement by HTA agencies. Clinical and Translational Science. 2021;14(4):1566–77.

24. Husereau D, Henshall C, Sampietro-Colom L, Thomas S. Changing health technology assessment paradigms? International journal of technology assessment in health care. 2016;32(4):191–9.

25. Lawlor R, Wilsdon T, Darquennes E, Hemelsoet D, Huismans J, Normand R, et al. Accelerating patient access to oncology medicines with multiple indications in Europe. Journal of market access & health policy. 2021;9(1):1964791.

26. Liberti L, Wang T. The regulatory-HTA decision-making interface: What the medical writer should know. Medical Writing. 2021;30:50–5.

27. Tafuri G, Pagnini M, Moseley J, Massari M, Petavy F, Behring A, et al. How aligned are the perspectives of EU regulators and HTA bodies? A comparative analysis of regulatory‐HTA parallel scientific advice. British journal of clinical pharmacology. 2016;82(4):965–73.

28. Wang T, McAuslane N, Liberti L, Leufkens H, Hövels A. Building synergy between regulatory and HTA agencies beyond processes and procedures—can we effectively align the evidentiary requirements? A survey of stakeholder perceptions. Value in Health. 2018;21(6):707–14.

29. Griffiths EA, Macaulay R, Vadlamudi NK, Uddin J, Samuels ER. The role of noncomparative evidence in health technology assessment decisions. Value in Health. 2017;20(10):1245–51.

30. Gozzo L, Romano GL, Romano F, Brancati S, Longo L, Vitale DC, et al. Health Technology Assessment of Advanced Therapy Medicinal Products: Comparison Among 3 European Countries. Frontiers in pharmacology. 2021;12.

31. Pinilla-Dominguez P, Naci H, Osipenko L, Mossialos E. NICE’s evaluations of medicines authorized by EMA with conditional marketing authorization or under exceptional circumstances. International Journal of Technology Assessment in Health Care. 2020;36(4):426–33.

32. Liberti L, Stolk P, McAuslane N, Somauroo A, Breckenridge A, Leufkens H. Adaptive licensing and facilitated regulatory pathways: a survey of stakeholder perceptions. Clinical Pharmacology & Therapeutics. 2015;98(5):477–9.

33. Sendyona S, Odeyemi I, Maman K. Perceptions and factors affecting pharmaceutical market access: results from a literature review and survey of stakeholders in different settings. Journal of market access & health policy. 2016;4(1):31660.

34. Chabot I, Rocchi A. Oncology drug health technology assessment recommendations: Canadian versus UK experiences. ClinicoEconomics and outcomes research: CEOR. 2014;6:357.

35. Pichon-Riviere M, Soto N, Augustovski F, Garcia-Marti S, Sampietro-Colom L. Involvement of relevant stakeholders in health technology assessment development. Background Paper Edmonton: Health Technology Assessment International. 2017;

36. Nicod E. Why do health technology assessment coverage recommendations for the same drugs differ across settings? Applying a mixed methods framework to systematically compare orphan drug decisions in four European countries. The European Journal of Health Economics. 2017;18(6):715–30.

37. Visintin E, Tinelli M, Kanavos P. Value assessment of disease-modifying therapies for Relapsing-Remitting Multiple Sclerosis: HTA evidence from seven OECD countries. Health Policy. 2019;123(2):118–29.

38. Oortwijn W, Determann D, Schiffers K, Tan SS, van der Tuin J. Towards integrated health technology assessment for improving decision making in selected countries. Value in Health. 2017;20(8):1121–30.

39. Francois C, Zhou J, Pochopien M, Achour L, Toumi M. Oncology from an HTA and health economic perspective. In: Regulatory and Economic Aspects in Oncology. Springer; 2019. p. 25–38.

40. Gordon J, Stainthorpe A, Jones B, Jacob I, Hertel N, Diaz J, et al. Non-Price-Related Determinants of Value and Access for Novel Non-small Cell Lung Cancer Treatments: A Cross-Country Review of HTA Decision Making. PharmacoEconomics-Open. 2021;5(4):701–13.

41. Abbas N, Hasan SS, Curley L. Access to medicines-a systematic review of the literature. Research in Social and Administrative Pharmacy. 2020;16(9):1166–76.

42. Spinner DS, Birt J, Walter JW, Bowman L, Mauskopf J, Drummond MF, et al. Do different clinical evidence bases lead to discordant health-technology assessment decisions? An in-depth case series across three jurisdictions. ClinicoEconomics and outcomes research: CEOR. 2013;5:69.

43. Mauskopf J, Walter J, Birt J, Bowman L, Copley-Merriman C, Drummond M. Differences among formulary submission guidelines: implications for health technology assessment. International Journal of Technology Assessment in Health Care. 2011;27(3):261–70.

44. Kleijnen S, George E, Goulden S, d’Andon A, Vitré P, Osińska B, et al. Relative effectiveness assessment of pharmaceuticals: similarities and differences in 29 jurisdictions. Value in health. 2012;15(6):954–60.

45. Kleijnen S, Lipska I, Alves TL, Meijboom K, Elsada A, Vervölgyi V, et al. Relative effectiveness assessments of oncology medicines for pricing and reimbursement decisions in European countries. Annals of oncology. 2016;27(9):1768–75.

46. Riedel R, Repschläger U, Griebenow R, Breitkopf S, Schmidt S, Guhl A. International standards for health economic evaluation with a focus on the German approach. Journal of clinical pharmacy and therapeutics. 2013;38(4):277–85.

47. Kamusheva M, Vassileva M, Savova A, Manova M, Petrova G. An overview of the reimbursement decision-making processes in Bulgaria as a reference country for the middle-income European countries. Frontiers in Public Health. 2018;6:61.

48. Charlton V, Rid A. Innovation as a value in healthcare priority-setting: the UK experience. Social Justice Research. 2019;32(2):208–38.

49. Lebioda A, Gasche D, Dippel FW, Theobald K, Plantör S. Relevance of indirect comparisons in the German early benefit assessment and in comparison to HTA processes in England, France and Scotland. Health Economics Review. 2014;4(1):1–14.

50. Makady A, Ten Ham R, de Boer A, Hillege H, Klungel O, Goettsch W. Policies for use of real-world data in health technology assessment (HTA): a comparative study of six HTA agencies. Value in Health. 2017;20(4):520–32.

51. Makady A, van Veelen A, Jonsson P, Moseley O, D’Andon A, de Boer A, et al. Using real-world data in health technology assessment (HTA) practice: a comparative study of five HTA agencies. Pharmacoeconomics. 2018;36(3):359–68.

52. Cheung K, Evers S, De Vries H, Lévy P, Pokhrel S, Jones T, et al. Most important barriers and facilitators of HTA usage in decision-making in Europe. Expert review of pharmacoeconomics & outcomes research. 2018;18(3):297–304.

53. Kaló Z, Bodrogi J, Boncz I, Dózsa C, Jóna G, Kövi R, et al. Capacity building for HTA implementation in middle-income countries: the case of Hungary. Value in health regional issues. 2013;2(2):264–6.

54. Kawalec P, Malinowski KP, Trąbka W. Trends and determinants in reimbursement decision-making in Poland in the years 2013–2015. Expert Review of Pharmacoeconomics & Outcomes Research. 2018;18(2):197–205.

**Appendix 3: Detailed results for rounds 1 and 2**

*Percentage agreement (SA+A) per value statements for rounds 1 and 2*

| **HTA features** | *Percentage agreement per access dimensions* | | |
| --- | --- | --- | --- |
|  | **Availability** | **Time to patient access (timeliness)** | **Affordability** |
| *1.Presence of an independent HTA body* | Round 1: 52%  Round 2: 41% | Round 1: 44 %  Round 2: 47% | **✓**  Round 1: 74 %  Round 2: 79% |
| *2.Scientific advice provided to manufacturers ahead of commencement of formal HTA process by HTA bodies* | **✓✓**  Round 1: 80 %  Round 2: 89% | **✓**  Round 1: 74%  Round 2: 79% | **✓**  Round 1: 70 %  Round 2: 79% |
| *3.Introduction of parallel review process to streamline marketing authorisation and HTA* | Round 1: 64%  Round 2: 50% | **✓✓**  Round 1: 85 %  Round 2: 95% | Round 1: 42%  Round 2: 32% |
| *4.Clarity of evidentiary requirements for value assessment in HTA* | **✓**  Round 1: 68%  Round 2: 76% | **✓✓**  Round 1: 85%  Round 2: 95% | Round 1:62 %  Round 2:53 % |
| *5.Reliance on real-world evidence in HTA in case of limited, incomplete, immature, or early phase clinical evidence* | **✓✓**  Round 1: 76 %  Round 2: 88% | **✓✓**  Round 1: 85%  Round 2: 100% | Round 1: 44%  Round 2: 37% |
| *6.Stakeholder involvement during the HTA process* | Round 1: 50 %  Round 2: 47% | Round 1: 69 %  Round 2: 74% | Round 1: 58%  Round 2: 63% |
| *7.Harmonization of rules for HTA methodologies, evidentiary requirements, and procedures across HTA bodies and systems at supranational level* | **✓✓**  Round 1: 75%†  Round 2: 76%† | **✓✓**  Round 1: 81%  Round 2: 89% | Round 1: 46%  Round 2: 37% |
| *8.Coordination of HTA rules, methods and processes across national and regional level, if both co-exist* | **✓**  Round 1: 70 %  Round 2: 75% | **✓✓**  Round 1: 88%  Round 2: 94% | Round 1: 54 %  Round 2: 53% |
| *9. Explicit recognition of additional dimensions of benefit beyond clinical and/or economic evidence considered during the evaluation of health technologies* | **✓**  Round 1: 71%  Round 2: 75% | Round 1: 65%  Round 2: 72% | Round 1: 52%  Round 2: 53% |
| *10. Legally binding HTA recommendations to be implemented in the shortest possible timeframe during reimbursement negotiations* | **✓**  Round 1: 83%  Round 2: 65% | **✓✓**  Round 1: 77%  Round 2: 76% | Round 1: 58%  Round 2: 25% |
| *11. No reliance on “HTA referencing”* | Round 1: 64%  Round 2: 33% | Round 1: 68%  Round 2: 35% | Round 1:33 %  Round 2: 13% |
| *12. Agreed-upon timelines for the completion of HTA process* | Round 1: 52 %  Round 2: 63% | **✓**  Round 1: 48%  Round 2: 94% | Round 1: 26%  Round 2: 12%* |
| *13.Established procedures on how uncertainties resulting from submitted evidence are managed and resolved within an agreed-upon timeframe* | **✓**  Round 1: 65 %  Round 2: 94% | **✓✓**  Round 1: 88%  Round 2: 83% | Round 1: 33%  Round 2: 61% |

*Notes:*

1. **✓**: Agreement was reached amongst participants with a value statement within one of the rounds: strongly agree and agree ≥ 75% approved by qualified majority
2. **✓✓**: Agreement was reached amongst participants with a value statement within both rounds: strongly agree and agree ≥ 75% approved by qualified majority
3. * this statement reached a 76% of participants choosing that they are neutral (neither agree nor disagree) with the positive impact of this value dimension on affordability
4. None of the value dimensions were rejected by absolute majority (SD + D > 50%)
5. †: this statement was approved by absolute majority (SA > 50% and SD + D < 33.3%) in both round 1 and 2

*Central tendency and dispersion per value statements for rounds 1 and 2*

| **HTA features** | *Round 1* | | | | *Round 2* | | | | *Change between rounds* | | |  |
| --- | --- | --- | --- | --- | --- | --- | --- | --- | --- | --- | --- | --- |
|  | **Availability** | **Time to patient access (timeliness)** | **Affordability** | **Availability** | | **Time to patient access (timeliness)** | **Affordability** | **Availability** | | **Time to patient access (timeliness)** | **Affordability** | |
| *1.Presence of an independent HTA body* | Median: 2 | Median:3 | Median:2 | Median: 3 | | Median: 3 | Median: 2 | Median:1 | | No change | No change | |
|  | IQR: 2 | IQR:2 | IQR:2 | IQR: 1 | | IQR: 2 | IQR:0 | IQR: -1 | | No change | IQR:-2 | |
| *2.Scientific advice provided to manufacturers ahead of commencement of formal HTA process by HTA bodies* | Median: 2 | Median:2 | Median:2 | Median:2 | | Median: 2 | Median: 2 | No change | | No change | No change | |
|  | IQR:1 | IQR:2 | IQR:2 | IQR: 1 | | IQR: 1 | IQR: 0 | No change | | IQR:-1 | IQR:-2 | |
| *3.Introduction of parallel review process to streamline marketing authorisation and HTA* | Median:2 | Median:2 | Median:3 | Median: 2.5 | | Median: 2 | Median: 3 | Median: 0.5 | | No change | No change | |
|  | IQR: 2 | IQR: 1 | IQR: 1 | IQR: 2 | | IQR: 1 | IQR: 2 | No change | | No change | IQR:-1 | |
| *4.Clarity of evidentiary requirements for value assessment in HTA* | Median: 2 | Median: 2 | Median: 2 | Median: 2 | | Median: 2 | Median: 2 | No change | | No change | No change | |
|  | IQR: 2 | IQR: 1 | IQR: 2 | IQR: 1 | | IQR: 1 | IQR: 1 | IQR:-1 | | No change | IQR:-1 | |
| *5.Reliance on real-world evidence in HTA in case of limited, incomplete, immature, or early phase clinical evidence* | Median: 2 | Median: 2 | Median: 3 | Median: 2 | | Median: 2 | Median: 3 | No change | | No change | No change | |
|  | IQR: 1 | IQR: 1 | IQR: 1 | IQR: 0 | | IQR: 0 | IQR: 1 | IQR:-1 | | IQR:-1 | No change | |
| *6.Stakeholder involvement during the HTA process* | Median: 2.5 | Median: 2 | Median: 2 | Median: 3 | | Median: 2 | Median: 2 | Median: 0.5 | | No change | No change | |
|  | IQR: 1.5 | IQR: 1 | IQR: 1 | IQR: 1 | | IQR: 1 | IQR: 1 | IQR:0.5 | | No change | No change | |
| *7.Harmonization of rules for HTA methodologies, evidentiary requirements, and procedures across HTA bodies and systems at supranational level* | Median: 1 | Median: 2 | Median: 3 | Median: 1 | | Median: 2 | Median: 3 | No change | | No change | No change | |
|  | IQR: 1.5 | IQR: 1 | IQR: 2 | IQR: 1 | | IQR: 1 | IQR: 2 | IQR:-0.5 | | No change | No change | |
| *8.Coordination of HTA rules, methods and processes across national and regional level, if both co-exist* | Median: 2 | Median: 2 | Median: 2 | Median: 2 | | Median: 2 | Median: 2 | No change | | No change | No change | |
|  | IQR: 2 | IQR: 1 | IQR: 2 | IQR: 1.5 | | IQR: 1 | IQR: 2 | IQR:-0.5 | | No change | No change | |
| *9. Explicit recognition of additional dimensions of benefit beyond clinical and/or economic evidence considered during the evaluation of health technologies* | Median: 2 | Median: 2 | Median: 2 | Median: 2 | | Median: 2 | Median: 2 | No change | | No change | No change | |
|  | IQR: 2 | IQR: 2 | IQR: 2 | IQR: 1.5 | | IQR: 2 | IQR: 1 | IQR:0.5 | | No change | IQR:-1 | |
| *10. Legally binding HTA recommendations to be implemented in the shortest possible timeframe during reimbursement negotiations* | Median: 2 | Median: 2 | Median: 2 | Median: 2 | | Median: 2 | Median: 3 | No change | | No change | Median:-1 | |
|  | IQR: 0 | IQR: 0 | IQR: 0 | IQR: 1 | | IQR: 1 | IQR: 0.5 | IQR:-1 | | IQR:-1 | IQR:-0.5 | |
| *11. No reliance on “HTA referencing”* | Median: 2 | Median: 2 | Median: 3 | Median: 3 | | Median: 3 | Median: 3 | Median:- 1 | | Median:-1 | No change | |
|  | IQR: 2 | IQR: 2 | IQR: 1 | IQR: 1 | | IQR: 1 | IQR: 0 | IQR: -1 | | IQR: -1 | IQR:- 1 | |
| *12. Agreed-upon timelines for the completion of HTA process* | Median: 2 | Median: 2 | Median: 3 | Median: 2 | | Median: 2 | Median: 3 | No change | | No change | No change | |
|  | IQR: 1 | IQR: 1 | IQR: 1 | IQR: 1.5 | | IQR: 0 | IQR:0 | IQR: -0.5 | | IQR:-1 | IQR:-1 | |
| *13.Established procedures on how uncertainties resulting from submitted evidence are managed and resolved within an agreed-upon timeframe* | Median: 2 | Median: 2 | Median: 3 | Median: 2 | | Median: 2 | Median: 2 | No change | | No change | Median: -1 | |
|  | IQR: 2 | IQR: 1 | IQR: 1 | IQR: 0 | | IQR: 0 | IQR: 1 | IQR: -2 | | IQR:-1 | No change | |

Note: No value statements received strong agreement (median 1), disagreement (median 4) and strong disagreement (median 5) by the participants in any round.

*Group agreement: Gwet’s coefficient*

| **HTA features** | *Round 1* | | | *Round 2* | | |
| --- | --- | --- | --- | --- | --- | --- |
|  | **Availability** | **Time to patient access (timeliness)** | **Affordability** | **Availability** | **Time to patient access (timeliness)** | **Affordability** |
| *1.Presence of an independent HTA body* | 0.20  slight | 0.38  fair | 0.15  slight | 0.59  moderate | 0.06  slight | 0.42  moderate |
| *2.Scientific advice provided to manufacturers ahead of commencement of formal HTA process by HTA bodies* | 0.51  moderate | 0.46  moderate | 0.47  moderate | 0.64  substantial | 0.52  moderate | 0.69  substantial |
| *3.Introduction of parallel review process to streamline marketing authorisation and HTA* | -0.05  poor | 0.26  fair | 0.34  fair | -0.07  poor | 0.53  moderate | 0.09  slight |
| *4.Clarity of evidentiary requirements for value assessment in HTA* | 0.38  fair | 0.61  substantial | 0.29  fair | 0.04  slight | 0.62  substantial | 0.50  moderate |
| *5.Reliance on real-world evidence in HTA in case of limited, incomplete, immature, or early phase clinical evidence* | 0.54  moderate | 0.60  moderate | 0.43  moderate | 0.75  substantial | 0.47  moderate | 0.44  moderate |
| *6.Stakeholder involvement during the HTA process* | 0.05  slight | 0.45  moderate | 0.55  moderate | 0.08  slight | 0.64  substantial | 0.65  substantial |
| *7.Harmonization of rules for HTA methodologies, evidentiary requirements, and procedures across HTA bodies and systems at supranational level* | 0.52  moderate | 0.52  moderate | 0.37  fair | 0.31  fair | 0.49  moderate | 0.11  slight |
| *8.Coordination of HTA rules, methods and processes across national and regional level, if both co-exist* | 0.39  fair | 0.38  fair | 0.14  slight | 0.37  fair | 0.57  moderate | 0.02  slight |
| *9. Explicit recognition of additional dimensions of benefit beyond clinical and/or economic evidence considered during the evaluation of health technologies* | 0.43  moderate | 0.10  slight | 0.46  moderate | 0.37  fair | 0.24  fair | 0.46  moderate |
| *10. Legally binding HTA recommendations to be implemented in the shortest possible timeframe during reimbursement negotiations* | 0.70  substantial | 0.59  moderate | 0.51  moderate | 0.10  slight | 0.44  moderate | 0.67  substantial |
| *11. No reliance on “HTA referencing”* | 0.33  fair | 0.23  fair | 0.65  substantial | 0.59  moderate | 0.71  substantial | 0.71  substantial |
| *12. Agreed-upon timelines for the completion of HTA process* | 0.24  fair | 0.67  substantial | 0.70  substantial | 0.38  fair | 0.72  substantial | 0.75  substantial |
| *13.Established procedures on how uncertainties resulting from submitted evidence are managed and resolved within an agreed-upon timeframe* | 0.40  fair | 0.41  moderate | 0.65  substantial | 0.82  Almost perfect | 0.78  substantial | 0.61  substantial |

*Stability*

| **HTA features** | *Wilcoxon matched-pair signed rank test* | | |
| --- | --- | --- | --- |
|  | **Availability** | **Time to patient access (timeliness)** | **Affordability** |
| *1.Presence of an independent HTA body* | P=1  Z=1  N=17 | P=0.500  Z= 1.414  N=19 | P=1  Z=-1  N=19 |
| *2.Scientific advice provided to manufacturers ahead of commencement of formal HTA process by HTA bodies* | P=0.5  Z= 1.414  N=17 | P=1  Z=1  N=19 | P=1  Z= 0.577  N=19 |
| *3.Introduction of parallel review process to streamline marketing authorisation and HTA* | P=1  Z=-1  N=18 | P=0.5  Z= 1.414  N=19 | P=1  Z=-1  N=19 |
| *4.Clarity of evidentiary requirements for value assessment in HTA* | P=0.5  Z= 1.414  N=17 | P=1  Z= 0.038  N=19 | P=0.25  Z= -1.731  N=18 |
| *5.Reliance on real-world evidence in HTA in case of limited, incomplete, immature, or early phase clinical evidence* | P=1  Z= -0.577  N=17 | P=0.5  Z=0.641  N=19 | P=0.500  Z= -1.414  N=19 |
| *6.Stakeholder involvement during the HTA process* | P=1  Z=0  N=17 | P=1  Z= -0.513  N=19 | P=1  Z=0  N=19 |
| *7.Harmonization of rules for HTA methodologies, evidentiary requirements, and procedures across HTA bodies and systems at supranational level* | P=1  Z=1  N=17 | P=0.5  Z= 1.414  N=19 | P=1  Z=-1  N=19 |
| *8.Coordination of HTA rules, methods and processes across national and regional level, if both co-exist* | P=1  Z=1  N=16 | P=1  Z=1  N=17 | P=1  Z=-1  N=17 |
| *9. Explicit recognition of additional dimensions of benefit beyond clinical and/or economic evidence considered during the evaluation of health technologies* | P=0.5  Z= 1.413  N=16 | P=0.5  Z= 1.414  N=18 | P=1  Z=1  N=17 |
| *10. Legally binding HTA recommendations to be implemented in the shortest possible timeframe during reimbursement negotiations* | P=1  Z= -0.378  N=16 | P= 0.2656  Z= 1.342  N=16 | P= 0.0625  Z= -2.138  N=15 |
| *11. No reliance on “HTA referencing”* | P= 0.0625  Z= -2.138  N=15 | P= 0.1719  Z= -1.501  N=15 | P=1  Z= -0.116  N=13 |
| *12. Agreed-upon timelines for the completion of HTA process* | P= 0.1094  Z= 1.911  N=15 | P= 0.0020  Z= 3.109  N=17 | P=1  Z= -0.180  N=15 |
| *13.Established procedures on how uncertainties resulting from submitted evidence are managed and resolved within an agreed-upon timeframe* | P= 0.3984  Z= 1.076  N=16 | P= 0.1250  Z= -1.890  N=18 | P=0.0156  Z= 2.636  N=17 |

| **HTA features** | *Spearman correlation coefficient* | | |
| --- | --- | --- | --- |
|  | **Availability** | **Time to patient access** | **Affordability** |
| *1.Presence of an independent HTA body* | 0.9678  P< 0.001  high | 0.9083  P< 0.001  high | 0.9434  P< 0.001  high |
| *2.Scientific advice provided to manufacturers ahead of commencement of formal HTA process by HTA bodies* | 0.9430  P< 0.001  high | 0.9670  P< 0.001  high | 0.8507  P< 0.001  high |
| *3.Introduction of parallel review process to streamline marketing authorisation and HTA* | 0.9602  P< 0.001  high | 0.9575  P< 0.001  high | 0.9035  P< 0.001  high |
| *4.Clarity of evidentiary requirements for value assessment in HTA* | 0.9255  P< 0.001  high | 0.8668  P< 0.001  high | 0.7947  P< 0.001  high |
| *5.Reliance on real-world evidence in HTA in case of limited, incomplete, immature, or early phase clinical evidence* | 0.7564  P< 0.001  high | 0.7721  P< 0.001  high | 0.9221  P< 0.001  high |
| *6.Stakeholder involvement during the HTA process* | 1.0000  P< 0.001  high | 0.8239  P< 0.001  high | 0.9244  P< 0.001  high |
| *7.Harmonization of rules for HTA methodologies, evidentiary requirements, and procedures across HTA bodies and systems at supranational level* | 0.9891  P< 0.001  high | 0.9242  P< 0.001  high | 0.9825  P< 0.001  high |
| *8.Coordination of HTA rules, methods and processes across national and regional level, if both co-exist* | 0.9767  P< 0.001  high | 0.9575  P< 0.001  high | 0.9764  P< 0.001  high |
| *9. Explicit recognition of additional dimensions of benefit beyond clinical and/or economic evidence considered during the evaluation of health technologies* | 0.8297  P< 0.001  high | 0.7725  P=0.002  high | 0.9862  P=0.002  high |
| *10. Legally binding HTA recommendations to be implemented in the shortest possible timeframe during reimbursement negotiations* | 0.6580  P=0.056  low | 0.1552  P= 0.5660  low | 0.5079  P= 0.0533  low |
| *11. No reliance on “HTA referencing”* | 0.7114  P= 0.0029  low | 0.5236  P= 0.0452  low | -0.0309  P= 0.9201  low |
| *12. Agreed-upon timelines for the completion of HTA process* | 0.7050  P= 0.0033  low | 0.4876  P= 0.0471  low | 0.1967  P= 0.4822  low |
| *13.Established procedures on how uncertainties resulting from submitted evidence are managed and resolved within an agreed-upon timeframe* | 0.3817  P= 0.1446  low | 0.4669  P= 0.0508  low | 0.5508  P=0.0219  low |
